# Generation of Tumor-Specific Cytotoxic T Cells From Blood *via In Vitro* Expansion Using Autologous Dendritic Cells Pulsed With Neoantigen-Coupled Microbeads

**DOI:** 10.3389/fonc.2022.866763

**Published:** 2022-03-31

**Authors:** Adela Kiessling, Keerthana Ramanathan, Ola B. Nilsson, Luigi Notari, Stefanie Renken, Rolf Kiessling, Hans Grönlund, Stina L. Wickström

**Affiliations:** ^1^Department of Oncology-Pathology, Karolinska Institutet, Stockholm, Sweden; ^2^Department of Clinical Neuroscience, Karolinska Institutet, Stockholm, Sweden; ^3^NEOGAP Therapeutics AB, Stockholm, Sweden; ^4^Theme Cancer, Patient Area Head and Neck, Lung and Skin, Karolinska University Hospital, Stockholm, Sweden

**Keywords:** neoantigen, tumor-specific antigens, autologous tumor recognition, dendritic cell-mediated activation, personalized cancer immunotherapy

## Abstract

For the past decade, adoptive cell therapy including tumor-infiltrating lymphocytes, genetically modified cytotoxic lymphocytes expressing a chimeric antigen receptor, or a novel T-cell receptor has revolutionized the treatment of many cancers. Progress within exome sequencing and neoantigen prediction technologies provides opportunities for further development of personalized immunotherapies. In this study, we present a novel strategy to deliver *in silico* predicted neoantigens to autologous dendritic cells (DCs) using paramagnetic beads (EpiTCer beads). DCs pulsed with EpiTCer beads are superior in enriching for healthy donor and patient blood-derived tumor-specific CD8+ T cells compared to DC loaded with whole-tumor lysate or 9mer neoantigen peptides. A dose-dependent effect was observed, with higher EpiTCer bead per DC being favorable. We concluded that CD8+ T cells enriched by DC loaded with EpiTCer beads are tumor specific with limited tumor cross-reactivity and low recognition of autologous non-activated monocytes or CD8+ T cells. Furthermore, tumor specificity and recognition were improved and preserved after additional expansion using our Good Manufacturing Process (GMP)-compatible rapid expansion protocol. Phenotypic analysis of patient-derived EpiTCer DC expanded CD8+ T cells revealed efficient maturation, with high frequencies of central memory and effector memory T cells, similar to those observed in autologous expanded tumor-infiltrating lymphocytes. These results indicate that DC pulsed with EpiTCer beads enrich for a T-cell population with high capacity of tumor recognition and elimination, which are features needed for a T-cell product to be used for personalized adoptive cell therapy.

## Introduction

Immunotherapy, including immune-checkpoint inhibitors (ICI) and adoptive cell therapy (ACT), is one of the most prominent and fastest developing fields within cancer treatment. ACT encompasses transfer of tumor-infiltrating lymphocytes (TILs), T cells genetically altered with a TCR or a chimeric antigen receptor (CAR), and NK cells or dendritic cell (DC) vaccines. The usage of genetically modified CAR T cells has been proven very effective against CD19+ hematological cancers while TIL therapies have been proven beneficial in the treatment of several solid tumors (1–4). Unfortunately, the population that benefits from these specific therapies is relatively limited; thus, novel alternative approaches to cancer immunotherapy are needed. One option is to target tumor antigens specifically. However, early studies targeting tumor-associated antigens (TAAs), self-antigens that are overexpressed or have an altered expression pattern, using TAA-directed TCR-transduced T cells or TAA-loaded DC vaccine, reported limited clinical responses or induction of autoimmune toxicity, including cross-reactivity-induced death (5–7). Central and peripheral tolerance against the chosen TAA or loss of antigen presentation on the tumor cell surface likely account for the low clinical efficacy (6).

Personalized immunotherapy using tumor-specific antigens (TSA) has the potential to be an ideal therapy, which maximizes efficacy while minimizing toxicity. Somatic mutations occurring in tumor cells can lead to the generation of novel TSA, which potentially can be recognized as non-self, referred to as neoantigens, which may be presented as tumor-specific peptides by major histocompatibility complex (MHC) molecules on the tumor cell surface. These peptides can be recognized by the immune system without induction of tolerance or risk of “off-target” effects on healthy tissues. Neoantigens are therefore promising targets for ACT and/or DC vaccine-based therapies (8). Numerous clinical trials based on neoantigens have been conducted or are ongoing, including DC loaded with neoantigens or delivered as peptides or mRNA (9).

Neoantigens are identified using next-generation sequencing (NGS), e.g., whole-exome sequencing (WES). Tumor-specific mutations are identified by comparing tumor biopsies to healthy tissues (in general, peripheral blood mononuclear cells) followed by different predictions tools to select for expressed mutations generating a neoantigen, which can efficiently be presented on MHC class I. To date, neoantigen predictions have mainly been focusing on antigens binding to frequent/common MHC class I/HLA class I alleles (10, 11). Computerized MHC-binding neoantigen predictions are in general focusing on MHC-binding affinity, endogenous expression (RNA) and processing, and/or in combination with mass spectrometry (MS). Combining several parameters increases the likelihood to predict neoantigens that are presented on MHC class I on the tumor cell surface. However, to verify if the predicted mutant peptides are true neoantigens, functional T-cell screens are needed.

We have previously shown that it is possible to predict clinically relevant neoantigens from two melanoma patients using free online tools for *in silico* neoantigen prediction (12). Neoantigens were identified by screening for TIL reactivity against custom made 9–10mer peptides. In addition, a DC vaccine-based stimulation method, loading the autologous DC vaccine with mutant 9mer peptides, was established to stimulate tumor-specific CD8+ T cells from the patient’s blood. Custom-ordered dextramers, specific for each neoantigen, in combination with CD107a expression (a marker for degranulation), were used to detect neoantigen-specific T cells recognizing the autologous tumor cells. In addition, mass spectrometry was used to verify neoantigen expression on the autologous tumor cell surface.

Others have shown that the usage of longer peptide sequences, ~25mers, to capture all possible “processing variants” of the neoantigen, can be favorable (13–15). Loading these longer peptides or combining several 25mers into (tandem)minigenes onto autologous antigen-presenting cells (APCs) has been shown to trigger efficient autologous T-cell responses measured by cytokine production or activation markers such as 4-1BB or PD1 (13, 16, 17).

Furthermore, longer peptide sequences have also been shown to be favorable when designing therapeutic neoantigen-based vaccines. The longer sequences promote antigen uptake and processing by APC and thereby help to facilitate a stronger T-cell response (18, 19). Due to tumor heterogeneity, targeting one neoantigen can result in outgrowth of tumor cells expressing other neoantigen(s), indicating the importance of targeting multiple neoantigens to lower the risk of immune escape and ensure elimination of all tumor cells (20, 21). In addition, Aurisicchio et al. have shown the importance of targeting multiple neoantigens with a predicted high binding affinity, <50 nM, to induce a poly-specific and poly-functional T-cell response in mice after minigene vaccinations (22). One alternative to neoantigen-based vaccines are DC vaccines loaded with whole tumor lysate (TL), which has been proven effective for certain cancer types (23). Although tumor lysate will contain peptides from all proteins in the cells and therefore “dilute” the neoantigen peptides and their ability to induce an immune response, the usage of tumor lysate circumvents the work with neoantigen prediction and peptide/minigene production.

In the present study, we have investigated several ways to load DC with tumor antigens to enrich for blood-derived tumor-specific CD8+ T cells. To this end, different approaches of tumor antigen administration were compared including whole tumor lysate, neoantigen 9mer peptides, or *via* longer neoantigen peptide sequences coupled to paramagnetic beads (EpiTCer) (24). EpiTCer beads are a novel way of delivering *in silico* predicted and recombinantly expressed neoantigen peptides. EpiTCer beads are paramagnetic beads covalently coupled to a neoantigen protein (NAG), consisting of six 21mer neoantigen peptides linked sequentially to each other. DC were generated according to our established Good Manufacturing Process (GMP) protocol. We have previously used DC generated from this protocol, in the form of DC vaccinations, in combination with TIL in patients with metastatic melanoma (NCT01946373).

We observed that DCs loaded with EpiTCer beads were superior in activating blood-derived autologous CD8+ tumor-specific T cells compared to DC pulsed with other sources of tumor antigen. Tumor recognition was MHC class I antigen dependent. In addition, patient-derived CD8+ T cells enriched using DC pulsed with EpiTCer beads were tumor specific with limited recognition of healthy cells. Phenotypic analysis of DC-expanded CD8+ T cells revealed efficient maturation of the CD8+ T cells with high frequencies of central memory and effector memory T cells, similar to those observed in TIL. We believe that EpiTCer beads represent an efficient method to pulse neoantigens onto DC, which can either be used to enrich for tumor-specific CD8+ T cells from peripheral lymphocytes in ACT or as a DC vaccine.

## Materials and Methods

### Cells

Patient-derived melanoma cell line, ANRU tumor cells, were generated as previously published (12) and cultured in Roswell Park Memorial Institute (RPMI) with 10%–20% fetal bovine serum (FBS) (Life Technologies, Waltham, USA) supplemented with penicillin (100 U/ml) and streptomycin (100 µg/ml) (Life Technologies). ANRU CD8+ T cells and monocytes were acquired through leukapheresis fractions 2 and 5, respectively (2018/2254-32). Peripheral blood samples (anonymized blood donations from healthy adult donors) were purchased from Karolinska University Hospital Blood Bank. Peripheral blood mononuclear cells (PBMCs) were isolated from healthy donor buffy coats using density centrifugation with Ficoll^®^ Paque Plus (GE Healthcare). The healthy donor PBMCs were screened and selected on HLA-A2+ donors. Healthy-donor-derived CD8+ T cells and CD14^+^ monocytes were isolated from PBMCs using positive CD8+ T cells isolation kit or CD14^+^ microbeads, respectively (Miltenyi Biotec, Bergisch Gladbach, Germany), following the manufacturer’s instructions.

### Neoantigen Predictions

For the identification of tumor-specific variants used to design neoantigen proteins, one melanoma patient, acronym ANRU, tumor tissue, and healthy control cells were used. Exome sequencing and prediction and identification and verification of the neoantigens ETV6 and NUP210 were performed as previously described (12). For the newly predicted neoantigens, the bioinformatics system PIOR (Personalised Immuno-Oncology Ranking, Stockholm, Sweden), developed by NEOGAP Therapeutics AB, was used. PIOR identifies and ranks tumor-specific variants; exome fastq files were processed with fastqc (https://www.bioinformatics.babraham.ac.uk/projects/fastqc/) to generate quality control parameters. The files were then mapped using bwa (http://bio-bwa.sourceforge.net/). Resulting mapped reads were sorted and deduplicated using samtools (http://www.htslib.org/). Resulting alignments were processed by an ensemble of multiple aligners (Vardict-Java, VarScan, FreeBayes, and Samtools mpileup). The resulting variant calls were combined, and high confidence somatic variant candidates were extracted from the list and then annotated using VEP (https://www.ensembl.org/info/docs/tools/vep/index.html) to establish possible variant effects. Finally, the resulting variants were ranked and presented to the user for visual inspection and selection. Tools used for the bioinformatics analysis are summarized in **Supplementary Table S2**. The mutations found and used as neoantigen sequences loaded on the beads were all single-nucleotide variants (SNVs).

### Neoantigen Design

**Figure 1:**
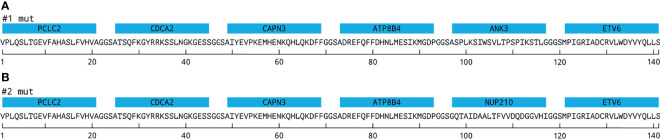
EpiTCer bead constructs. Neoantigen proteins containing six 21mer polypeptides interconnected *via* GGS linkers and covalently coupled to a paramagnetic bead, EpiTCer beads®. **(A)** Displays construct 1 (#1) containing indicated neoepitopes. **(B)** Displays construct 2 (#2) containing indicated neoepitopes. For additional gene, mutation, and sequence information, see *Materials and Methods* and **Supplementary Figure S1** and **Supplementary Tables S1, S2**.

Novel neoantigen proteins (NAGs) were designed as recombinant construct genes. Top-ranked tumor-specific variants identified by PIOR were assembled with the previously validated neoantigens from ETV6 and NUP210, forming ANRU NAG #1 and #2, respectively (see **Figure 1**). Corresponding wild-type (WT) constructs were also generated, replacing the mutated codons with the corresponding WT ones (see **Supplementary Figure S1**). The nucleotide sequences were optimized for expression in *E. coli*; flanking *Bsa*I sites were added to the optimized DNA sequences and directionally cloned into a modified pET28 vector (Merck-Millipore) as previously described (25). When expressed in the modified pET28 vector, the NAG is flanked by a polylysine coupling tail containing K residues flanked by GGS linkers and a W residue at the N-terminus and an eight-histidine purification tag at the C-terminus.

### Neoantigen Production

The cloned constructs were transformed into BL21-AI *Escherichia coli* (Thermo Fisher Scientific, Waltham, USA), and the NAGs were expressed and purified as described (26), using a modified elution buffer with a pH of 2.0. The eluted and equilibrated NAGs were analyzed for purity by sodium dodecyl sulfate–polyacrylamide gel electrophoresis (SDS-PAGE) as described, and their concentration was measured using a nanophotometer (Implen, Munich, Germany).

### EpiTCer Beads

The NAGs were covalently coupled to 1 µm paramagnetic polystyrene beads [Sera-Mag™ Carboxylate-Modified Magnetic Beads (SpeedBeads, Marlborough, MA, USA), Cytiva], utilizing covalent coupling of primary amines in the neoantigens to the carboxylic groups present on the beads, after EDC/NHS activation, as described (26). Still reactive, non-coupled carboxylic groups were deactivated using a 50-mM Bicine buffer. For endotoxin removal and normalization purposes, the coupled beads were conditioned by four washes with sterile filtered 2M NaOH, followed by four washes with endotoxin-free sterile Dulbecco’s phosphate-buffered saline (DPBS) pH 7.4 containing 0.1% Poloxamer 188 (a surfactant that decreases potential beads aggregation). For quality control, NAG load was evaluated by staining coupled beads with Ni-NTA Atto 488 compound, which targets the C-terminal 8His tag on the neoantigens (Sigma-Aldrich). NAG load in the beads was evaluated using flow cytometry (Guava EasyCyte-Luminex Corporation, Saint Louis, USA) and the InCyte analysis software. The number of molecules per bead was calculated to range between 2 and 6 million, based on protein quantification by means of bicinchoninic acid (BCA) assay, flow cytometric determination of bead concentrations, and predicted molecular weight of the purified NAG.

### Generation of Neoantigen-Specific CD8+ T Cell From the Blood

Patient- or healthy donor-derived CD14+ monocytes were matured into DC vaccines as previously described (2, 27). Shortly, CD14+ monocytes were cultured in Cellgro^®^ (Cellgenix, Freiburg, Germany) supplemented with IL-4 (20 ng/ml) and granulocyte-macrophage colony-stimulating factor (GM-CSF) (100 ng/ml) (Cellgenix or Peprotech) for 48 h into immature DC (imDC). imDC were harvested and loaded with indicated antigen/neoantigen source (tumor lysates healthy donor, 30 ng/ml; ANRU, 30 μg/ml and beads at indicated bead/DC ratio) and further matured into mature DC by 18 h culture in Cellgro supplemented with IL-4 (20 ng/ml), GM-SCF (100 ng/ml), interferon-gamma (IFNγ) (1,000 IU/ml, Imukin^®^, Boehringer Ingelheim, Ingelheim, Germany), R848 (2.5 μg/ml, InVivogen, San Diego, US), Poly I:C (20 μg/ml, InVivogen, San Diego, US), and lipopolysaccharide (LPS) (10 ng/ml, InVivogen, San Diego, US). DCs loaded with neoantigen 9mer ETV6, and NUP210, and mature DCs were pulsed with 10 μg/ml (JPT peptides) for 30 min at 37°C and washed before use in co-culture with CD8+ T cells. The DCs were co-cultured with autologous CD8+ T cells in a 1:5 ratio for 10–14 days in CellGro^®^ supplemented with 2% human AB serum (Karolinska University Hospital Blood Bank) and 20 IU/ml IL-2 (Proleukine, Novartis, Basil, Switzerland). All expansions were performed in 96-well U-bottom plates (Cornigen, Corning, New York, USA) if nothing else is stated.

### Tumor Lysates

Tumor lysates were generated using our GMP protocol (2, 27).

### Flow Cytometry and Functional Assays

All antibodies and FACS reagents were used according to the manufacture’s recommendation, if not otherwise stated. All antibodies were titrated for optimal signal-to-noise ratio. Samples were fixed with 2% paraformaldehyde (PFA) (Thermo Scientific) for 15 min before acquisition on a NovoCyte (ACEA Biosciences, San Diego, USA). Compensation was performed using AbC™ Total Antibody Compensation Bead Kit and ArC™ Amine Reactive Compensation Bead Kit (both Invitrogen). FlowJo Software (TreeStar) was used for analysis. All staining protocols included a dead cell marker (LIVE/DEAD^®^ fixable Aqua Dead cell stain (Invitrogen).

T cells were analyzed for anti-CD8 (clone SK1, APC-Cy7), anti-CD3 (clone UCHT1, PE-Cy7), anti-CD45RA-AF488 (clone CI100) (all from BioLegend, San Diego, USA), anti-CCR7-AF647 (clone 3D12, BD), and HLA-A2 (clone BB7.2, PE, BioLegend).

*Degranulation/CD107a.* Shortly, all long-term co-cultures were harvested and counted, and CD8+ T cells were re-stimulated using ANRU tumor cells, ratio of 1:5, or indicated 9mer peptide (ETV6 or NUP210) 10 μg/ml (JTP peptides). Detection of activated tumor-specific T cells was performed by staining with CD107a (clone H4A3, FITC, BioLegend) antibody, which was added to stimulated T-cell cultures at experiment setup (12, 28). GolgiPlugTM and GolgiStopTM (BD Bioscience) were added after 2 h co-culture, and cells were harvested after an additional 4 h co-culture, harvested and stained for neoantigen specificity (neoantigen specific dextramers, see above) and/or cell surface markers (see above). In experiments where intracellular staining was performed, cells were stained for dead cells, then CD3 and CD8, before fixation and permeabilization using CytoPerm/CytoFixTM (BD Biosciences) and intracellular staining for IFN-γ (clone 4S.B3, PE, BioLegend). When indicated, MHC class I interactions were blocked on ANRU tumor cells for 30 min at 37°C with 20 µg/ml anti-HLA-ABC antibody (clone W6/32, BioLegend) before addition of CD8+ T cells.

## Results

### Neoantigen Delivery Using EpiTCer^®^ Beads

EpiTCer beads consist of paramagnetic beads in 1 μm size range, onto which neoantigen proteins are covalently coupled. Each construct, comprising six neoantigen polypeptides (21mer), spaced with a three amino acid flexible GGS linker, were coupled to the paramagnetic beads. Previous studies have shown that particles of this size facilitate phagocytosis and efficient antigen processing and presentation by the engulfing antigen-presenting cell (24, 29–31). In the present study, two different versions of EpiTCer beads have been used, harboring on their surface two different constructs (construct 1 and construct 2), containing in silico-predicted T-cell neoantigens derived from whole exome sequencing from one HLA-A0201 melanoma patient, acronym ANRU (**Figures 1A, B**; **Supplementary Table S1**). The ETV6 and NUP210 neoantigens were previously discovered and validated as 9mers (**Figure 1**) (12).

### Efficient Stimulation of Blood-Derived CD8+ T Cells Using EpiTCer Beads® Pulsed DC

To validate the concept of administrating neoantigens to DC through EpiTCer beads, the capability of EpiTCer-loaded DC to stimulate autologous tumor-specific CD8+ T cells from blood was investigated. To this end, HLA-A2+ healthy donor-derived CD14+ monocytes and CD8+ T cells were isolated, and monocytic DC were generated according to our GMP protocol (27). ANRU-derived EpiTCer beads were pulsed onto the immature DC during the second maturation step. ANRU-derived tumor lysate (TL) loaded DC were used as control. For EpiTCer beads, several bead/DC ratios were used. To analyze the efficacy of the tumor antigen-loaded DC to stimulate T cells, long-term co-cultures using autologous DC and blood derived CD8+ T cells was performed. Enriched tumor-specific CD8+ T cells were harvested and re-stimulated with the same melanoma tumor cell line (ANRU) from which the neoantigens and the tumor lysate were derived. Tumor recognition was measured by degranulation (CD107a expression) and cytokine production (IFNγ) using flow cytometry.

**Figure 2:**
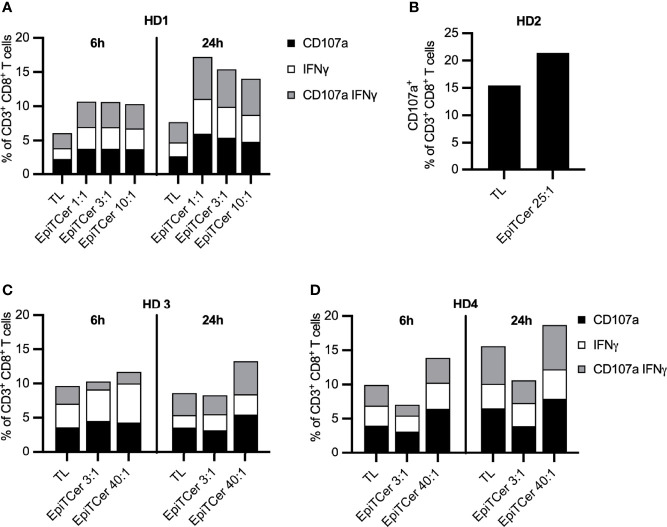
Efficient stimulation of blood-derived CD8+ T cells using EpiTCer^®^ bead pulsed DC. HLA-A2+ healthy donor (HD) blood-derived CD14+ monocytes and CD8+ T cells were isolated; monocytes matured into imDC were loaded with indicated source of ANRU derived tumor antigens and further matured into DC. Long term co-cultures with DC and CD8+ T cells were performed and T cells harvested and re-stimulated with ANRU tumor cells. Tumor recognition was measured by CD107a expression and IFNγ production using flow cytometry. **(A–D)** HD CD8+ T cells were co-cultured with DC pulsed ANRU tumor lysate (TL) or EpiTCer beads #1 at indicated bead/DC ratio. Tumor specificity was measured by re-stimulation with ANRU tumor cells and measured by CD107a expression **(B)** or CD107a expression and IFNγ production **(A**, **C**, **D)**, using flow cytometry. Each donor represents one independent experiment.

We observed that the DC loaded with EpiTCer beads were more potent in enriching and stimulating tumor-specific CD8+ T cells compared to tumor lysate loaded DC, for each of the 1:1–10:1 bead/DC ratios used (**Figure 2A**). A higher EpiTCer bead:DC ratio of 40:1 could further increase tumor recognition and induce an efficient CD8+-mediated antitumoral response for three additional healthy donors (**Figures 2B–D**).

Furthermore, 24-h re-stimulation of the EpiTCer activated CD8+ T cells with ANRU tumor cells resulting in a more efficient tumor recognition than the 6-h re-stimulation, at all tested bead/DC ratios, as measured by degranulation (CD107a) and IFNγ production. In contrast, CD8+ T cells stimulated with DC loaded with tumor lysate, only a marginal difference between 24 vs. 6 h ANRU tumor re-stimulation was noted (**Figure 2**).

Thus, we conclude that DCs loaded with EpiTCer beads, carrying several *in silico* predicted neoantigens, are more efficient in enriching for and activating tumor-specific CD8+ T cells from the blood, compared to tumor-lysate-loaded DC.

### Efficient Neoantigen Delivery Through Autologous DC Loaded With EpiTCer^®^ Beads

Next, we investigated which method of tumor antigen loading confers the best capacity of DC to stimulate autologous tumor-specific CD8+ T cells. Healthy-donor-derived DCs were loaded either with several ratios of EpiTCer beads, with tumor lysate or as negative control DC without antigen source (MOCK). As a comparison, DCs pulsed with two validated ANRU neoantigen 9mer peptides, ETV6 and NUP210 (12), which were also included in the EpiTCer constructs, were used. After 14 days of stimulating CD8+ T cells with antigen-loaded DC, tumor reactivity was analyzed by re-stimulation of CD8+ T cells with ANRU tumor cells or with the ETV6/NUP210 9mer neoantigen peptides (only for T cell that had been co-cultured with DC pulsed with the corresponding 9mer peptide). Tumor reactivity was measured by CD107a expression using flow cytometry.

**Figure 3:**
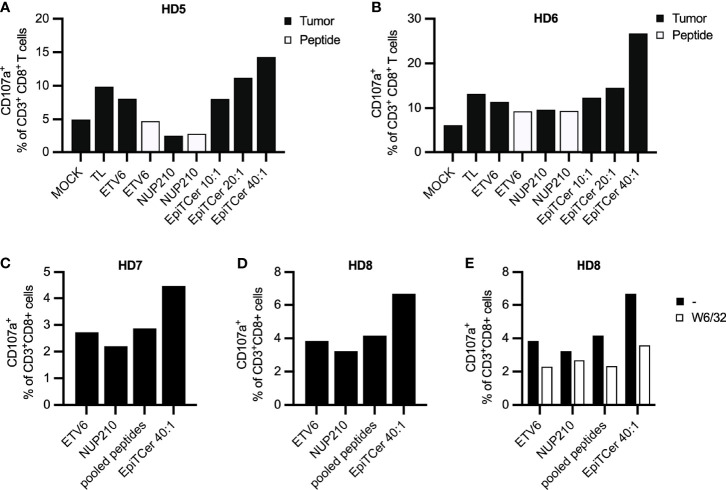
Efficient neoantigen delivery through autologous DC loaded with EpiTCer^®^ beads. HLA-A2+ healthy donor (HD) blood-derived CD14+ monocytes and CD8+ T cells were isolated; monocytes matured into imDC were loaded with indicated source of ANRU derived tumor antigens and further matured into DC. Long-term co-cultures with DC and CD8+ T cells were performed; T cells was harvested and re-stimulated with ANRU tumor cells or 9mer neoantigen peptides. T-cell activation was measured by CD107a expression using flow cytometry. **(A, B)** HD CD8+ T cells were co-cultured with DC pulsed ANRU tumor lysate (TL), indicated 9mer neoantigen peptide or EpiTCer beads #2 at indicated bead/DC ratio. Tumor specificity or T-cell activation was assessed by re-stimulation with ANRU tumor cells or indicated 9mer neoantigen peptide, respectively. **(C–E)** HD CD8+ T cells were co-cultured with DC pulsed with indicated 9mer neoantigen peptide (separately or combined/pooled) or EpiTCer beads #2 at indicated bead/DC ratio. Tumor specificity was assessed by re-stimulation with ANRU tumor cells with **(E)** or without **(C, D)** the presence of MHC class I blockade (W6/32). Values in panels **(D, E)** without W6/32 (−) were obtained within the same experiment. Each donor represents one independent experiment.

In both healthy donors, an EpiTCer bead/DC ratio dose-dependent increase in the frequency of CD107+ tumor reactive CD8+ T cells was observed (**Figures 3A, B****)**. In addition, the highest EpiTCer bead/DC ratio (40:1) generated and triggered an increased frequency of tumor-specific CD8+ T cells when compared to DC loaded with either neoantigen 9mer peptides or tumor lysate. However, DC loaded with either neoantigen 9mer peptides, ETV6 or NUP210, or tumor lysate induced increased frequencies of tumor reactive CD8+ T cells when compared to the unloaded DC, MOCK (**Figures 3A, B****)**. Notably, re-stimulation with ANRU tumor cells triggered a stronger T-cell activation than re-stimulation with the neoantigen 9mer peptide, ETV6, in CD8+ T cells previously enriched by DC loaded with the same peptide (**Figures 3A, B****)**. This indicates that loading of neoantigen peptides on MHC class I on CD8+ T cells can trigger activation alone but not as strong as when combined with other potential tumor antigens providing co-stimulatory signals expressed on the tumor cells.

Next, we assessed if loading the DC with pooled ETV6 and NUP210 peptides could increase the enrichment for tumor-specific T cells to a similar extent as the one observed with EpiTCer-beads-loaded DC. Notably, there was no additive effect on tumor recognition when stimulating CD8+ T cells with DC loaded with ETV6 combined with NUP210 (**Figures 3C, D****)**. In both healthy donors, EpiTCer-loaded DC induced an increased frequency of CD8+ tumor-specific T cells compared to ETV6 and NUP210 peptides delivered separately or in pools. Comparable results were observed in a third donor, showing no additional effect by pooling ETV6 and NUP210 (**Supplementary Figure S2A**). These results indicate that there is a dominant neoantigen peptide/epitope, “masking” the response towards additional neoantigens. Furthermore, the dominant neoantigen peptide differs between the healthy donors (HD) investigated, with ETV6 or NUP210 being the dominant epitope in HD 7 and 8 (**Figures 3C, D****)** or HD 10 (**Supplementary Figure S1A**), respectively.

To assess MHC class I antigen-dependent tumor cell recognition, CD8+ T cells stimulated with DC pulsed with EpiTCer beads, ETV6 or NUP210 9mer peptides separately or pooled, were re-stimulated with ANRU tumor cells with and without MHC class I blocking antibody (W6/32). All CD8+ T-cell-mediated tumor recognition was MHC class I-antigen dependent, with the strongest influence of MHC class I antigen-dependent presentation observed in the DC pulsed with EpiTCer beads stimulated CD8+ T-cell population (**Figure 3E**).

### Efficient Expansion of Patient Blood-Derived Tumor-Specific CD8+ T Cells Using DC Loaded With EpiTCer Beads^®^

To investigate the clinical relevance of DC loaded with EpiTCer beads, autologous ANRU DC, blood-derived CD8+ T cells, and ANRU tumor cell line were used. ANRU imDC were loaded with tumor lysate, ETV6 or NUP210 9mer peptides, or several ratios of EpiTCer beads. Non-coated EpiTCer beads (empty, E) and EpiTCer beads coated with the corresponding wild-type (WT) sequences were used as controls. Longtime co-cultures of DC-CD8+ T cells were performed, followed by re-stimulation with autologous ANRU tumor cells. Tumor recognition was measured by degranulation (CD107a expression) and IFNγ production analyzed by flow cytometry.

**Figure 4:**
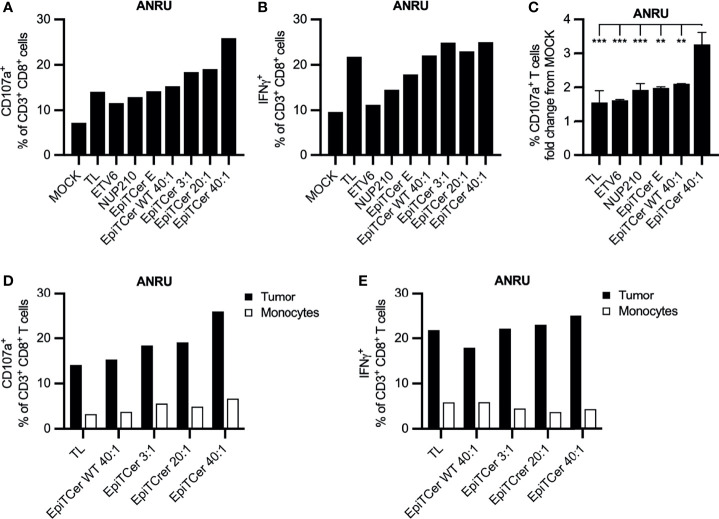
Efficient expansion of patient blood-derived tumor-specific CD8+ T cells using DC loaded with EpiTCer beads®. ANRU blood-derived CD14+ monocytes and CD8+ T cells were isolated; monocytes matured into imDC were loaded with indicated source of ANRU derived tumor antigens and further matured into DC. Long-term co-cultures with DC and CD8+ T cells was performed; T cells were harvested and re-stimulated with ANRU tumor cells or ANRU non-activated monocytes. Tumor recognition or healthy cell reactivity was measured by CD107a expression or IFNγ production using flow cytometry. **(A, B)** ANRU CD8+ T cells were co-cultured with DC pulsed ANRU tumor lysate (TL), indicating 9mer neoantigen peptide, non-coated EpiTCer beads (empty, E), EpiTCer beads carrying the corresponding wild-type sequence (WT), or EpiTCer beads #2. EpiTCer beads were used at indicated bead/DC ratio. Tumor specificity was assessed by re-stimulation with ANRU tumor cells. **(C)** ANRU CD8+ T-cell tumor recognition, presenting values from **Figures 4A** and **5A** and **Supplementary Figure S2B**. **(D, E)** ANRU CD8+ T cells were co-cultured as described in panels **(A, B)**, and T-cell activation was measured by re-stimulation with ANRU tumor cells or non-activated ANRU monocytes. Values in panels **(A, D)** and **(B, D)** for ANRU tumor reactivity were obtained within the same experiment. **(C)** Statistical analysis one-way ANOVA, Tukey’s multiple comparison test. Definition of significance: ***p < 0.001, **p < 0.01.

In three independent experiments, DC loaded with EpiTCer beads induced the highest frequency of CD107a+ autologous tumor-specific CD8+ T cells (**Figures 4A, C** and **Supplementary Figure S2B**). In addition, all EpiTCer bead/DC ratios induced a higher frequency of CD107a+ CD8+ T cells compared to more traditional ways to pulse DC with antigens, such as custom made 9mer neoantigen peptides, ETV6 and NUP210, or whole tumor lysate. Furthermore, CD8+ T cells stimulated with EpiTCer beads pulsed DC also displayed the most efficient cytokine production upon ANRU re-stimulation, although the difference to DC loaded with tumor lysate was less than for CD107a (**Figure 4B**). When comparing tumor recognition between all conditions, CD8+ T cells enriched by EpiTCer pulsed DC had a significantly increased frequency of tumor-specific T cells (**Figure 4C**). Notably, all DC conditions, including the control EpiTCer beads and non-coated and wild-type beads, were better at enriching for tumor-specific CD8+ T cells compared to unloaded DC (MOCK). This was the case independently of measuring degranulation or cytokine production (**Figures 4A, B****)**. This indicates that loading of DC with EpiTcer beads, independently of antigen coupled, was able to stimulate DC to become more efficient in activating blood-derived CD8+ T cells, although this activation was not MHC class I antigen dependent (data not shown).

Most of predicted neoantigens have a single amino acid mutation, while the rest of the sequence remains identical to the wild type. One can argue that the risk of expanding self-reactive T-cell populations may be higher when using longer neoantigen sequences, allowing alternative processing, compared to using pre-determined 9mer peptides. In addition, the EpiTCer beads are coupled to six 21mer long neoantigens interconnected with GGS linkers. Although the linker is designed to not to be similar to any sequence expressed in humans, they could potentially, due to alternative processing of the sequence by the DC, be at risk of stimulating self-reactive T cells. Therefore, the self-reactivity of the T-cell product was investigated by re-stimulating the autologous DC-activated CD8+ T-cell populations with autologous monocytes or CD8+ T cells as target cells.

None of the DC loaded with tumor lysate, EpiTCer beads or EpiTCer WT bead, stimulated CD8+ T cell populations displayed an efficient recognition of the autologous monocytes, measured by degranulation or IFNg production (**Figures 4D, E****)**. In addition, there was no recognition by the DC-stimulated CD8+ T cells when exposed to autologous non-stimulated CD8+ T cells (data not shown). This shows that DCs loaded with either tumor lysate or any of the EpiTCer bead concentrations do not react to the healthy cells investigated, indicating that the established stimulation method to enhance tumor-specific CD8+ T cells from the blood could potentially be used for adoptive cell therapy. However, further examination of its safety is needed. Comparable results were observed using healthy-donor-derived DC and CD8+ T cells (**Supplementary Figure S2C**).

### EpiTCer-Loaded Autologous DCs Enrich for Tumor-Specific CD8+ T Cells Do Not Show Tumor Cross-Reactivity

**Figure 5:**
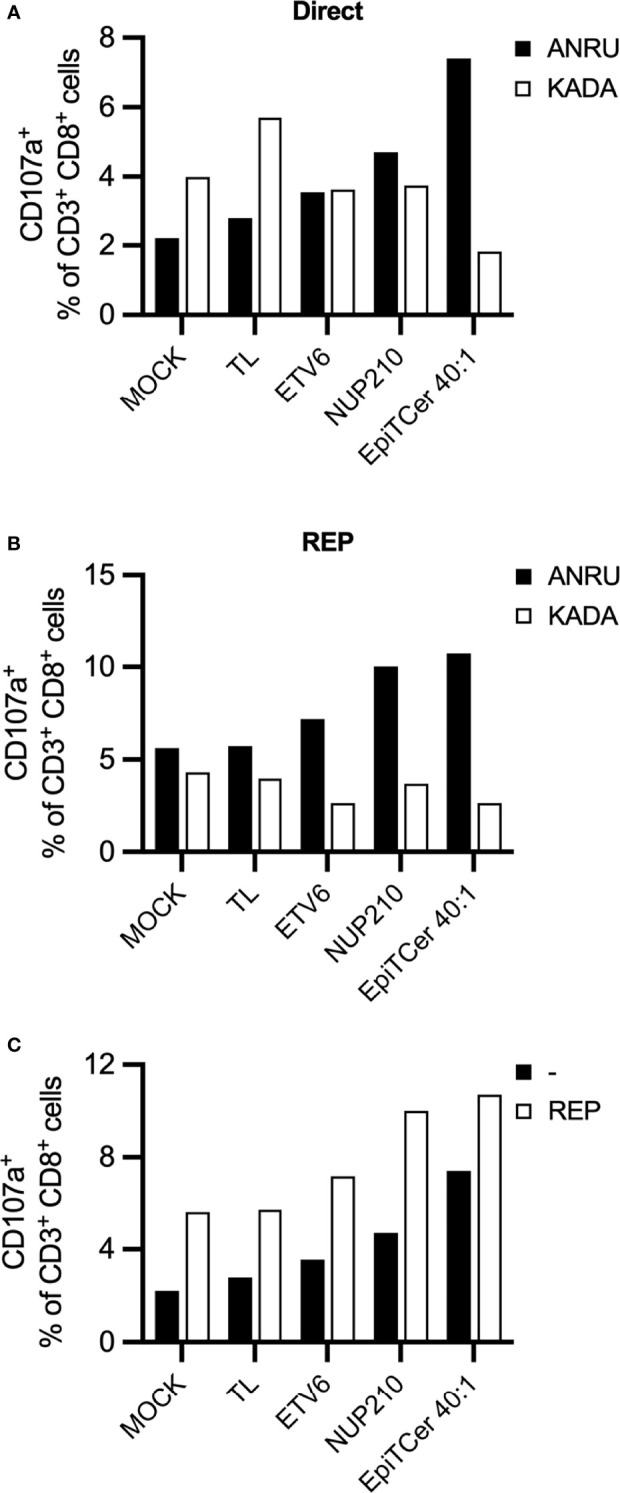
EpiTCer-loaded autologous DC enrich for highly tumor-specific CD8+ T cells with limited tumor cross-reactivity. ANRU blood-derived CD14+ monocytes and CD8+ T cells were isolated; monocytes matured into imDC were loaded with indicated source of ANRU-derived tumor antigens and further matured into DC. Long-term co-cultures with DC and CD8+ T cells were performed: T cells harvested and **(A)** re-stimulated with indicated tumor cell line **(B)** further expanded *via* rapid expansion (REP). Tumor recognition was measured by CD107a expression. **(A)** ANRU CD8+ T cells were co-cultured with DC-pulsed ANRU tumor lysate (TL), indicating 9mer neoantigen peptide or EpiTCer beads #2 at indicated bead/DC ratio. Tumor specificity was assessed by re-stimulation with autologous ANRU tumor cells or allogenic KADA (HLA-A2+ melanoma cell line). **(B)** DC-enriched ANRU CD8+ T cells were further expanded using a REP protocol, harvested and re-stimulated with autologous ANRU tumor cells or allogenic KADA tumor cells. Panel **(C)** shows the CD8+ mediated ANRU tumor recognition observed before (−) and after REP (REP); same values as in panels **(A, B)** are displayed.

To assess tumor specificity of DC-stimulated ANRU CD8+ T cell products, autologous ANRU DCs were loaded with tumor lysate, neoantigen 9mer peptides (ETV6 or NUP210) or EpiTCer beads. The DC-stimulated CD8+ T cells were (1) challenged with either autologous ANRU tumor cells or an allogenic melanoma cell line KADA, both HLA-A2+, or (2) further expanded using a GMP-compatible rapid expansion (REP) protocol (2). When directly re-stimulated with either the autologous ANRU or allogenic KADA tumor cells, the CD8+ T cell expanded with EpiTCer-beads-loaded DC displayed a very high tumor selectivity compared to all other stimulation conditions (**Figure 5A**). Notably, CD8+ T cells stimulated with DC MOCK or tumor lysate had an increased reactivity against the allogenic tumor cell line compared to the autologous cell line (**Figure 5A**). When re-stimulating the different REP-expanded CD8+ T cell populations with ANRU or KADA tumor cells, a preserved tumor selectivity was observed (**Figure 5B**). When comparing ANRU recognition with or without rapid expansion, we observed an increased tumor reactivity after REP (**Figure 5C**). These results indicate that tumor selectivity is preserved and enhanced during unspecific stimulation using anti-CD3 and radiated feeder cells.

### EpiTCer Pulsed DCs Efficiently Induce Functional Maturation of Blood-Derived CD8+ T Cells

**Figure 6:**
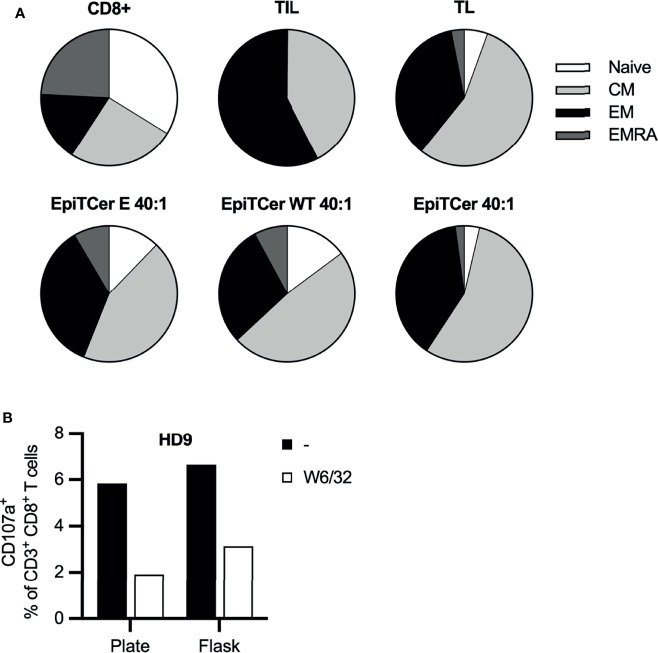
EpiTCer-pulsed DC efficiently induces functional maturation of blood-derived CD8+ T cells. ANRU **(A)** or healthy donor **(B)** blood-derived CD14+ monocytes and CD8+ T cells were isolated, and imDC were loaded with indicated source of ANRU-derived tumor antigens and matured into DC. Long-term co-culture with DC and CD8+ T cells was performed. T cells were harvested and **(A)** phenotypic analysis was performed or **(B)** ANRU tumor reactivity was measured. **(A)** Long-term co-culture with ANRU CD8+ T cells and ANRU DC tumor lysate (TL), non-coated EpiTCer beads (empty, E), EpiTCer wide-type beads (WT) or EpiTCer beads at 40:1 bead/DC ratio was performed. CD8+ T cells were harvested, and phenotypic analysis of maturation status was performed using flow cytometry. Cells were gated on lymphocytes/single cells/live cells/CD3+CD8+ cells, and maturation was investigated *via* CCR7 and CD45RA. For gating strategy, see **Supplementary Figure 3**. **(B)** HD CD8+ T cells were long-term co-cultured with DC loaded with ANRU tumor lysate. Co-culture was performed in plate (96w plates) or in cell culture flasks; CD8+ T cells were harvested and re-stimulated with ANRU tumor cells with and without MHC class I blocking (W6/32). HD, each donor represents one independent experiment.

Phenotypic analysis of the long-term DC-stimulated autologous ANRU blood-derived CD8+ T cells was performed to analyze their expression of various maturation and memory markers. Non-DC stimulated ANRU CD8+ T cells and ANRU TIL, expanded using our clinical trial protocol, were used as controls. DC pulsed with EpiTCer beads or tumor lysate efficiently induced maturation of CD8+ T cells into central and effector memory T cells, with a phenotype similar to the one observed for the CD8+ T cells derived from the TIL. CD8+ T cells stimulated with DC loaded with empty EpiTCer beads or EpiTCer beads coated with wild-type sequences displayed a less mature phenotype (**Figure 6A**).

To further investigate the possibility of using DC loaded with EpiTCer beads for expansion of tumor-specific T cells as a cell product for adoptive cell therapy, large-scale expansion was explored.

Healthy donor-derived CD8+ T cell and EpiTCer-loaded DC were used to compare the tumor specificity/reactivity when CD8+ T cells were expanded in small or large scale, using plates or cell culture flasks, respectively. A similar expansion of tumor-specific T cells was observed independently of expansion setup, with a trend of an increased tumor recognition if CD8+ T cells were expanded in large scale using cell culture flasks (**Figure 6B**). Furthermore, blocking MHC class I-mediated recognition using monoclonal antibodies revealed that tumor recognition was antigen dependent for both cell products.

## Discussion

In this study, we have demonstrated that EpiTCer beads are an efficient method of loading *in silico* predicted and recombinantly expressed 21mer neoantigens onto autologous DC to enrich for and stimulate tumor-specific CD8+ T cells from peripheral lymphocytes. We observed that autologous DC pulsed with EpiTCer beads were more efficient/significantly better in stimulating both healthy-donor- and patient-derived tumor-specific CD8+ T cells from the blood compared to tumor antigens delivered *via* neoantigen 9mer peptides or tumor lysate (**Figures 1** and **4A**). Tumor specificity was assessed by recognition of the autologous ANRU tumor cell line, from which the neoantigens and the tumor lysate were predicted or generated, respectively. Furthermore, tumor recognition by healthy-donor-derived CD8+ T cells expanded by DC pulsed with tumor antigens, especially when delivered *via* EpiTCer beads, was reduced upon MHC class I blocking (**Figures 3E** and **6C**).

Numerous methods have previously been used to verify and enrich for tumor and/or neoantigen-specific T cells. In addition, several methods of analyzing, detecting, and defining recognition of tumor and/or neoantigen-specific T cells have been employed. This includes neoantigen-specific tetramers (32, 33) or selection based on activation/antigen-experienced markers, such as PD1+ or 4-1BB+ (13, 17, 34–36). In addition, peripheral lymphocytes recognizing oncogenes, p53 and KRAS, derived neoantigens have been investigated (15, 37, 38). Furthermore, healthy-donor-derived peripheral lymphocytes have been investigated as a source of neoantigens-specific T cells to be used for therapy (39, 40).

We and others have previously shown that it is possible to predict and identify neoantigens that can be used to generate autologous tumor and neoantigen-specific T cells from patient-derived tumor TIL and/or peripheral lymphocytes (12, 17, 32, 34–36). Several other studies have shown that it is possible to identify neoantigens to generate neoantigen-specific T cells within TIL or peripheral lymphocytes populations (13, 15, 33, 37–40). In these studies, neoantigen specificity was assessed by measuring the T cells capability to recognize or become activated upon re-stimulation with the corresponding neoantigen-loaded DC, with neoantigen pulsed/transduced tumor cells or with an allogeneic tumor cell line containing the specific mutation. We have previously shown that recognition of neoantigen-derived peptides does not necessarily mean efficient recognition of autologous tumor cells (12), displaying the importance of assessing recognition of the autologous tumor cells. In the Tumor Neoantigen Selection Alliance (TESLA) consortium, it was found that only 6% of the neoantigen peptides, top ranked by each participants algorithm, were able to bind to the patient’s HLA alleles and form a multimeric complex. To our knowledge, no efforts were done to analyze if the T cells recognizing these peptides were also able to kill the autologous tumors from which they were derived (41, 42).

We observed a relatively high tumor reactivity by the CD8+ T cells enriched by non-coated and wild-type EpiTCer beads (**Figure 4**). This could be explained by the size, ~1 μm, of the EpiTCer beads themselves, which was chosen based on previous studies displaying increased capacity to stimulate antigen processing and presentation (24, 29–31). Non-coated EpiTCer and wild-type beads generated very similar levels of tumor-reactive T cells, indicating that the wild-type sequence or the linker regions did not further enhance the stimulatory capacity.

Furthermore, peripheral tumor-specific ANRU CD8+ T cells enriched using autologous DC loaded with EpiTCer beads, ANRU tumor lysate, or EpiTCer wild-type beads, displayed limited recognition of autologous healthy tissue/cells, monocytes, and unstimulated CD8+ T cells. These results indicate that alternative processing of either the neoantigen sequences or the linker regions does not produce peptides that stimulate autoreactive T cells. The implications from these findings are however limited, and to further exclude the risk of autoreactivity of the T cells, a more extensive screening of a panel of normal tissues will have to be performed. Unfortunately, there were too few CD8+ T cells enriched *via* non-coated EpiTCer-pulsed DC to analyze healthy tissue cross-reactivity.

In line with previously published reports (13, 32), an efficient distinction between autologous, ANRU, and allogeneic KADA tumor cells was observed in all DC-enriched conditions (**Figure 5**). Furthermore, the tumor specificity was maintained during rapid expansion, with an increased autologous tumor recognition (**Figures 5B, C****)**.

It has previously been demonstrated that naive CD8+ T cells within peripheral lymphocyte populations can have reduced effector functions, low proliferation, and low TCR avidity, and are unlikely to engage in TCR–antigen interactions when compared to memory T cells, T_CM_ and T_EM_ (43, 44). Central memory T cells (T_CM_) have been shown to possess a greater capacity to persist *in vivo*, while effector memory T cells (T_EM_) have immediate effector functions although with lower proliferative capability (45, 46). We observed that DC loaded with EpiTCer beads were more efficiently matured CD8+ peripheral lymphocytes into central and effector memory T cells, when compared to DC loaded with non-coated EpiTCer or wild-type beads (**Figure 6A**). These results are consistent with the tumor recognition analysis and with the evidence that EpiTCer-loaded beads are efficient in expanding a T-cell population with high capacity of tumor recognition and elimination, as measured by CD107a expression.

The data presented in this study strongly support the potential of using EpiTCer-beads-loaded DC as a method for enriching for patient-derived tumor-specific T cells from peripheral blood and/or as a DC vaccination approach. Neoantigen-loaded therapeutic DC vaccines have been shown to enrich for pre-existing neoantigen-specific T cells *in vivo* in patients with cutaneous melanoma or advanced lung cancer (9, 47). It has also been shown that peptide/mRNA-based neoantigen vaccines can stimulate neoantigen-specific T-cell populations *in vivo*, leading to improved clinical outcome in melanoma patients (48, 49). However, there are also clinical trials showing a limited or no beneficial effect of neoantigen-based vaccines as monotherapy or in combination with checkpoint inhibitors (50–54). These reports indicate that cancer/neoantigen vaccines cannot completely eradicate the disease (55). Most likely, cancer vaccines will have to be combined with approaches targeting the immune suppressive microenvironment to eliminate MDSC and T regs and other suppressive mechanisms, thereby allowing the effector functions of tumor-specific CD8+ T cells. It is likely that DC, when optimally activated with methods such as loading them with beads, may be able to activate both tumor-specific T cells and non-specific immune mechanisms.

These questions have been addressed in mouse models with conflicting results. Salvatori et al. investigated different therapeutic combinations of checkpoint inhibitors (ICI), α-CTLA4 or α-PD1, and neoantigen-based cancer vaccines (56). They found that the combination of α-CTLA4 and a neoantigen vaccine had a large impact on tumor growth, while monotherapy using α-CTLA4 had no effect on tumor growth using a CT26 tumor model. In addition, the combination of α-CTLA4 and a neoantigen vaccine significantly reduced MC38 tumor growth, while the addition of α-PD1 had no effect on tumor growth. However, Li et al. investigated neoantigen-specific T cells in combination with α-CTLA4 or α-PD1 in Lewis lung carcinoma and observed an expansion of neoantigen-specific CD8+ TIL after ICI therapy but no effect on tumor regression (57). When combining a neoantigen vaccine with α-CTLA4 and α-PD1 therapies, a specific expansion of neoantigen-specific CD8+ TIL was detected but no effect on tumor growth. These results indicated the complexity of designing an efficient treatment protocol and how it should be evaluated. The presented study is built on neoantigens predicted from patient material. We have investigated the option of performing *in vivo* elimination assays based on inoculation of immune compromised NSG mice. These experiments were done with ANRU tumor cells to assess tumor elimination/regression after injection of autologous CD8+ T cells stimulated with DC loaded with the different antigen sources. Unfortunately, ANRU tumor was found to grow poorly in NSG mice, and *in vivo* elimination assays could not therefore be performed.

To date, the neoantigen prediction methods have mainly been focusing on MHC class I-binding peptides, and therefore, CD8+ T-cell responses and the usage of neoantigen-specific T cells for ACT has been skewed towards investigating the effect mediated by CD8+ T cells. However, longer, 15-30mer, neoantigen peptides incorporating a 9–11mer CD8+ T-cell neoantigen peptide can also serve as a neoantigen presented on MHC class II for CD4+ T cells. This has been demonstrated in clinical trials exploring neoantigen vaccination where a clear CD4+ T-cell dominated response has been observed (38, 49). In line with other studies targeting neoantigens to produce a T-cell product for ACT, we focused on the CD8+ T-cell mediated antitumoral response. However, Arbelaez et al. showed that vaccination utilizing nanoparticle-delivered neoantigen peptides stimulated both a CD4+ and CD8+ T-cell-mediated response with a CD8+ T-cell-dependent antitumoral response (58). In contrast, vaccination with naked peptides only triggered a CD4+ T-cell response without any antitumoral effect (59). These results encourage further investigations asking if EpiTCer-bead-delivered neoantigens can trigger a stronger antitumoral effect if a combined CD4+ and CD8+ T cell response is initiated. Furthermore, Wei et al. have shown that formation of a neoantigen cancer vaccine consisting of polymerized synthetic peptides through a reversible polycondensation reaction resulted in an improved antigen delivery to lymph nodes (LN) and facilitated efficient activation of antigen-presenting cells compared to a naked peptide vaccine (58). The advanced formula for rapid release of neoantigen peptides in response to intracellular reduction activity upon internalization facilitated the delivery of peptide antigens and enabled cross-presentation by APC, triggering an increased CD8+ T-cell-mediated response and CD8+ T-cell maturation. In the present study, six different 21mer neoantigen polypeptides, each comprising 6 neoepitopes, were coupled separately to paramagnetic beads. The covalent linking of the designed neoantigen constructs offers several benefits. Directional coupling *via* the polylysine domain is a feature for direct conjugation, and the eight-histidine tag is used for purification and coupling quantification. In addition, the EpiTCer bead size induces activation and natural antigen presentation when phagocytosed by APC. Yet, the coupling method is not hampered by inaccurate disulfide bonds. Taken together, the covalent linking allows efficient coupling of the diverse repertoires of personalized neoantigens. In accordance with Wei et al., an efficient CD8+ T-cell maturation and antitumoral response was observed when delivering neoantigen peptides *via* EpiTCer beads.

To our knowledge, we have shown here for the first time an efficient enrichment of tumor-specific CD8+ peripheral lymphocytes using DC loaded with personalized neoantigens delivered *via* paramagnetic beads. We suggest that a novel therapy using *in vitro* expanded tumor-specific peripheral blood-derived T cells using EpiTCer-pulsed DC combined with a DC vaccine could be a possible option for patients with inoperable tumors and could be considered for patients with tumor types that do not allow efficient TIL production.

## Data Availability Statement

The original contributions presented in the study are included in the article/**Supplementary Materials**. Further inquiries can be directed to the corresponding author.

## Ethics Statement

The studies involving human participants were reviewed and approved by the Local Ethics Committee Stockholm, Sweden (no. 2015/18-62-32). The patients/participants provided their written informed consent to participate in this study.

## Author Contributions

AK, KR, SR, and SLW. carried out the experiments and data analysis. SLW designed the experiments. ON performed neoantigen predictions, and LN produced the neoantigen proteins and coupling to EpiTCer beads. SLW wrote the manuscript with input from all the co-authors. HG and RK helped supervise the project. HG, RK, and SLW conceived the original idea. SLW supervised the project. All authors contributed to the article and approved the submitted version.

## Funding

SLW was supported by Karolinska Institutet (2-5586/2017), RK was supported by grants from the Swedish Cancer Society (190104Pj01H and 190108Us01H), the Cancer Society in Stockholm (194123), the Swedish Medical Research Council (2019-01212), and Stockholm City Council Project Grant (LS 2018-1157).

## Conflict of Interest

SLW, RK, and TCER Oncology AB have a patent application for this invention. SLW is affiliated to NEOGAP Therapeutics and receives a research grant from this company. HG is the founder and co-owner and receives a research grant from the company NEOGAP Therapeutics AB, which holds patents and pending patents regarding the EpiTCer platform. ON and LN were employed by NEOGAP Therapeutics AB. RK is a Scientific Advisor for Anocca AB and Phio Pharmaceutics and receives research grants from these companies.

The remaining authors declare that the research was conducted in the absence of any commercial or financial relationships that could be construed as a potential conflict of interest.

## Publisher’s Note

All claims expressed in this article are solely those of the authors and do not necessarily represent those of their affiliated organizations, or those of the publisher, the editors and the reviewers. Any product that may be evaluated in this article, or claim that may be made by its manufacturer, is not guaranteed or endorsed by the publisher.
