# Supplementary material for: Generation of Tumor-Specific Cytotoxic T Cells From Blood via In Vitro Expansion Using Autologous Dendritic Cells Pulsed With Neoantigen-Coupled Microbeads
Source: Front Oncol. 2022 Mar 31;12:866763. doi: 10.3389/fonc.2022.866763 (PMC9009257; doi:10.3389/fonc.2022.866763)
Supplement: Supplementary file 1 [file Presentation_1.pdf]

Suppl. Figure 1

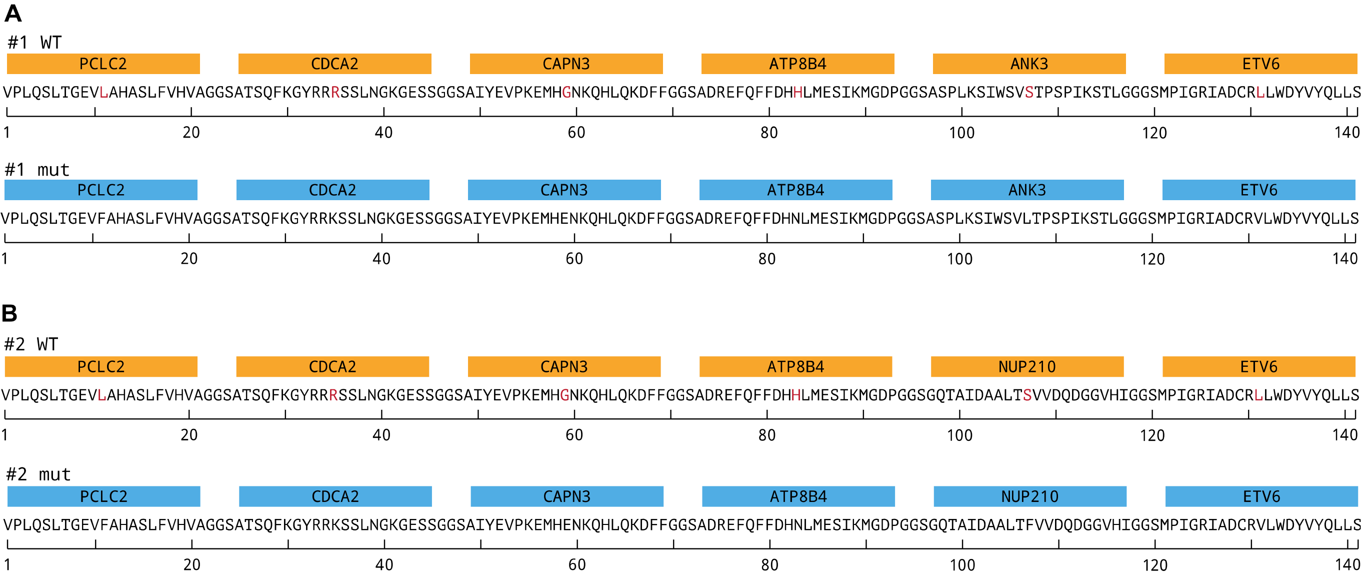

Supplementary Figure 1, EpiTCer bead constructs;
Neoantigen proteins containing six 21mer polypeptides interconnected via GGS linkers and covalently coupled to a paramagnetic bead, EpiTCer beads. (A) Displays construct 1 (#1) containing indicated neoepitopes and corresponding wild type sequences. (B) Displays construct 2 (#2) containing indicated neoepitopes corresponding wild type sequences. For additional gene, mutation and sequence information, see materials and methods and supplementary, supplementary table 1 and 2.

Suppl. Figure 2

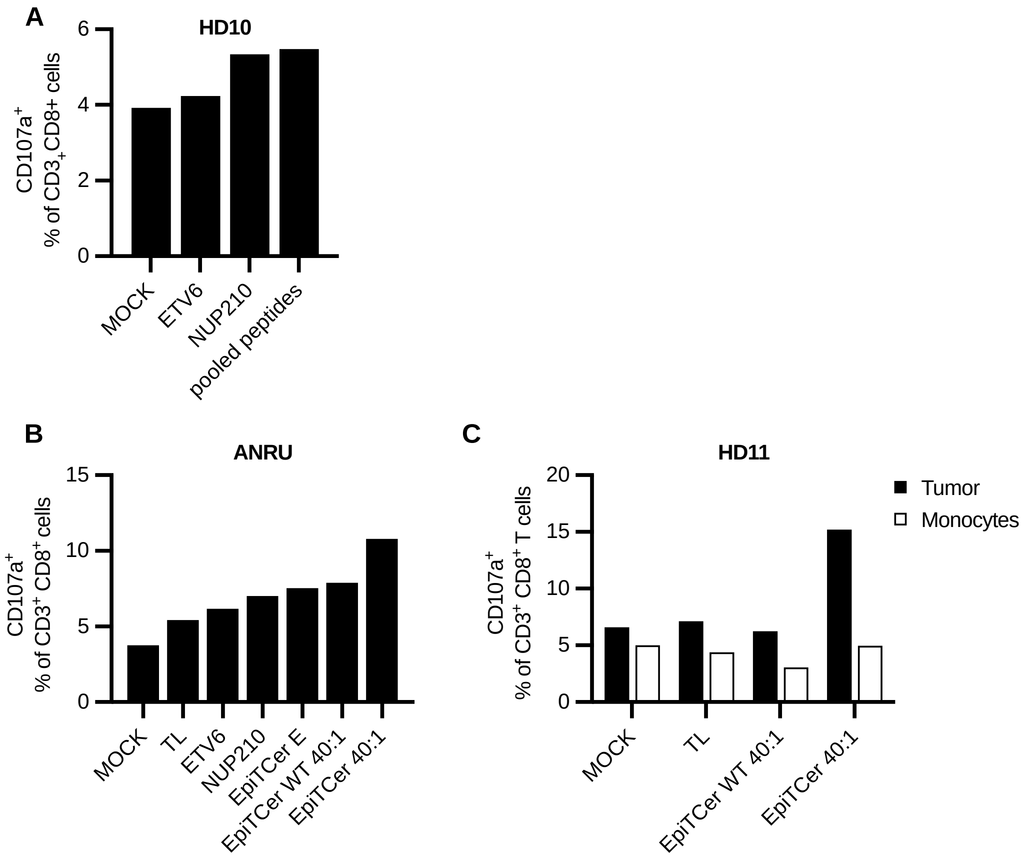

Supplementary Figure 2:

Healthy donor or ANRU blood derived CD14+ monocytes and CD8+ T cells were isolated, monocytes matured into imDC were loaded with indicated source of ANRU derived tumor antigens and further matured into DC. Long term co-cultures with DC and CD8+ T cells was performed, T cells harvested and re-stimulated with ANRU tumor cells or autologous non-activated monocytes. Tumor recognition or healthy cell reactivity was measured by CD107a expression using flow cytometry. (A) Healthy donor derived CD8+ T cells were co-cultured with DC pulsed with 9 mer neoantigen peptides, ETV6 or NUP210, either separately of combined (pooled). MOCK, DC without antigens were used as a control. (B) ANRU CD8+ T cells were co-cultured with DC pulsed ANRU tumor lysate (TL), indicated 9mer neoantigen peptide, non-coated EpiTCer beads (E, empty), EpiTCer beads carrying the corresponding wild type sequence (WT) or EpiTCer beads #2. (C) Healthy donor derived CD8+ T cells were co-cultured with DC pulsed with tumor lysate (TL), EpiTCer beads carrying the corresponding wild type sequence (WT) or EpiTCer beads #2. MOCK, DC without antigens were used as a control. CD8+ T cells were re-stimulated using ANRU tumor cells or autologous monocytes.

Suppl. Figure 3

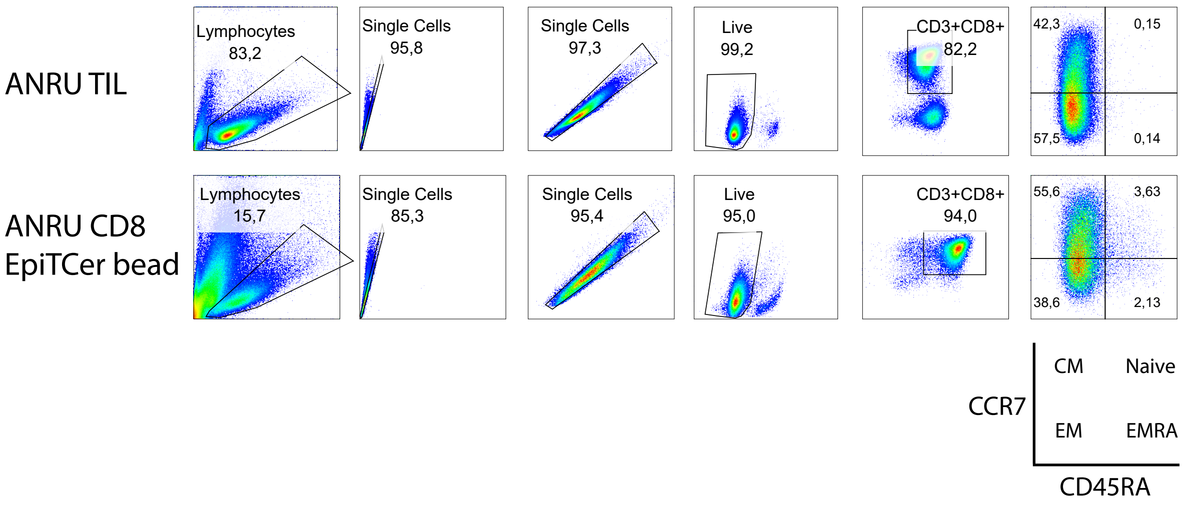

Supplementary Figure 3, CD8+ T cell maturation efter EpiTCer pulsed DC stimulation
ANRU blood derived CD14+ monocytes and CD8+ T cells were isolated, and imDC were loaded with EpiTCer bead, ratio 40:1, and matured into DC. Long term co-culture with DC and CD8+ T cells was performed. T cells were harvested and phenotypic analysis was performed using flow cytometry. Cells were gated on lymphocytes/single cells/live cells/CD3+CD8+ cells and maturation was investigated via CCR7 and CD45RA. ANRU TIL was used for comparison.

Supplementary table 1

Supplementary table 1: pedicted neoantigen sequences and genes

Variant ID	Variant Effect	Gene	Mutated Peptide
chr3:17011951:C:T	L486F	PLCL2	VPLQSLTGEVFAHASLFVHVA
chr8:25507659:G:A	R998K	CDCA2	ATSQFKGYRRKSSLNGKGESS
chr15:42402801:G:A	G467E	CAPN3	AIYEVPKEMHENKQHLQKDFF
chr15:49934113:GATG:AATT	H452N	ATP8B4	ADREFQFFDHNLMESIKMGDP
chr10:60076250:G:A	S1544L	ANK3	ASPLKSIWSVLTPSPIKSTLG
chr12:11884453:C:G	L340V	ETV6	MPIGRIADCRVLWDYVYQLLS
chr3:13376304:G:A	S427F	NUP210	GQTAIDAALTFVVDQDGGVHI

Supplementary table 2

Table 2. Bioinformatics tools used by the PIOR bioinformatics pipeline.

Function	Tool
Reference Genome	GRCh38
Reference Proteome	gencode v35 & RefSeq v109.20200815
Quality Control	fastqc v0.11.9, mosdepth v0.2.9, custom scripts
Mapping	bwa v0.7.17
Filtering / deduplication	Samtools v1.10
Copy number variation	cnvkit v0.9.7
Variant Effect Prediction	snpeff v5.0
Variant calling ensemble	Vardict-Java v1.8.2: Tumor-Normal Caller
	Vardict-Java v1.8.2: Single Library Caller
	VarScan v2.4.4
	FreeBayes v1.3.2
	Samtools v1.10: Mpileup
